# Successful Ultrasound-Guided Superficial Cervical Plexus Block Treatment for Head and Neck Pain with an Unusually Delayed Onset Following Ventriculoperitoneal Shunt: A Case Report

**DOI:** 10.3390/medicina59111909

**Published:** 2023-10-28

**Authors:** Ching-Yuan Hu, Ying-Zhen Huang, Sheng-Tzung Tsai, Po-Kai Wang

**Affiliations:** 1 Department of Anesthesiology, Hualien Tzu Chi Hospital, Buddhist Tzu Chi Medical Foundation, Hualien 97004, Taiwan; 101311110@gms.tcu.edu.tw (C.-Y.H.); 108324115@gms.tcu.edu.tw (Y.-Z.H.); 2 School of Medicine, Tzu Chi University, Hualien 97004, Taiwan; flydream@tzuchi.com.tw; 3 Department of Neurosurgery, Hualien Tzu Chi Hospital, Buddhist Tzu Chi Medical Foundation, Hualien 970, Taiwan

**Keywords:** ventriculoperitoneal shunt, complication, head and neck pain, shunt catheter malposition, nerve block

## Abstract

***Background and Objectives:*** Ventriculoperitoneal (VP) shunt placement is the most common treatment for cerebrospinal fluid diversion. Head and neck pain occurring after a long period following VP shunt insertion is rarely reported. Here, we present a rare case of head and neck pain occurring 2 years after surgery due to irritation of the superficial cervical plexus by the VP shunt. ***Case Description:*** A 46-year-old female patient received VP shunt placement surgery. Two years after the surgery, she experienced a left temporal headache with neck pain on the left side, which extended to the left para-auricular and fascial region. Ultrasound (US) scanning revealed that the VP shunt passed within the superficial cervical fascia and through the left sternocleidomastoid muscle (SCM). Additionally, friction of the branches of the superficial cervical plexus and of the greater auricular and lesser occipital nerves caused by the VP shunt was found underneath the lateral border of the SCM. Subsequently, the blocking and hydro-release of the left superficial cervical plexus were performed. After four series of treatments, the patient’s head and neck pain vanished, and the frequency of the headaches was substantially reduced. The patient was regularly followed-up in the outpatient department of neurosurgery. ***Conclusions:*** Head and neck pain caused by the malpositioning of a VP shunt catheter with an unusually delayed onset is a rarely reported complication and could be easily neglected. Patients with head and neck pain following VP shunt insertion should be checked using US scanning to identify the potential origin of the pain and receive adequate treatments. Intraoperative US-guided tunnelling is suggested to avoid the malpositioning of the VP shunt catheter.

## 1. Introduction

Cerebrospinal fluid (CSF) shunting through ventriculoperitoneal (VP) shunt placement remains the primary neurosurgical operation for patients with hydrocephalus [1,2]. Although the VP shunt provides an effective diversion of CSF and improves the patient’s life quality, numerous problems may come after the shunt’s placement. The complications of the VP shunt’s placement include infections, over-drainage, kinking, disconnection, malpositioning of the catheter tip, catheter obstruction, subdural hematoma, abdominal pseudocyst, and bowel perforation [2,3,4]. Head and neck pain occurring after a long period following VP shunt insertion is rarely reported and may easily be dealt with through symptomatic treatment. Here, we present a rare case of head and neck pain with an unusually delayed onset due to the irritation of the superficial cervical plexus by the VP shunt. We describe the approaches utilized to diagnose and treat the pain.

## 2. Case Description

**Figure 1:**
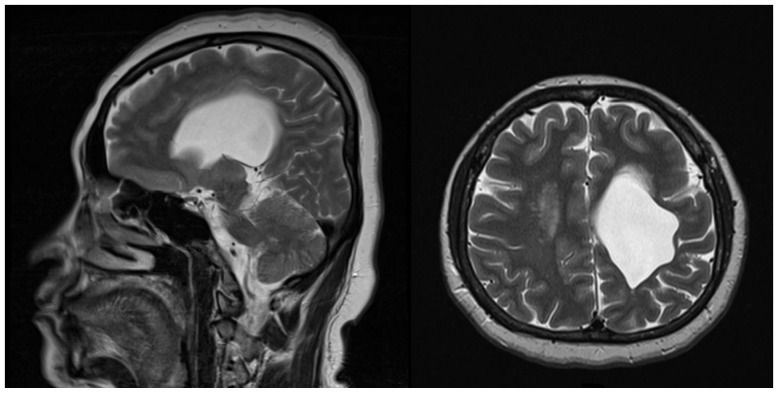
Brain magnetic resonance imaging revealing one huge cystic lesion (60 × 45 × 40 mm^3^) within left lateral ventricle.

**Figure 2:**
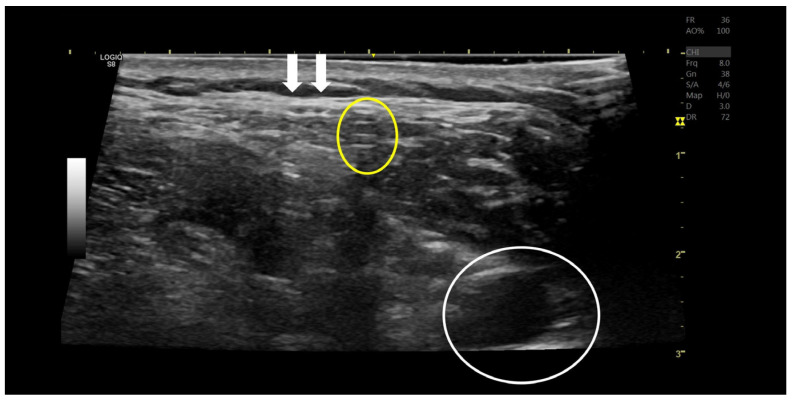
Ultrasonography of left cervical region. White arrows: branches of the superficial cervical plexus; yellow circle: ventriculoperitoneal (VP) shunt tube; white circle: internal carotid artery. The ultrasonography shows that the VP shunt passed within the superficial cervical fascia and through the left sternocleidomastoid muscle (SCM).

**Figure 3:**
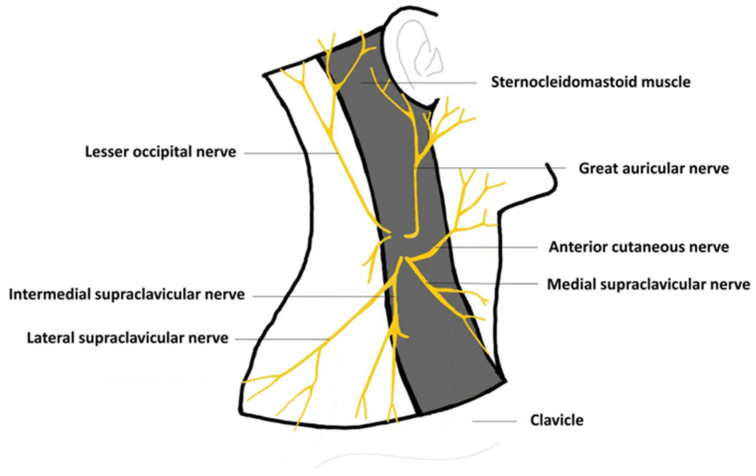
Schematic illustration showing the branches of superficial cervical plexus. Modified from: Talamantes et al. *Anesth. Crit. Care* 2021, 3, 21–28 [5].

A 46-year-old female patient had a huge brain cystic lesion (60 × 45 × 40 mm^3^) within the left lateral ventricle, with gradually unsteady gait and muscle weakness on the right side (Figure 1). She successfully received VP shunt (STRATA valves shunt, Medtronic, Minneapolis, MN, USA) placement surgery for the drainage of the excess fluid without postoperative complications. After the surgery, the right-side weakness improved, and her gait became steady without dizziness or headache. Two years after the VP shunt surgery, she was admitted to our hospital due to a headache. She had no recent history of fever, nausea, vomiting, symptoms of increased intracranial pressure, unsteady gait, lethargy, or trauma events. She had hypertension as the only comorbidity and regularly took anti-hypertension medications. She complained that she frequently experienced intermittent left temporal headache with left neck pain, which extended to the left para-auricular and fascial regions. A similar pain pattern could be induced with palpation over the left lateral cervical region of the patient. The pain was not relieved when the head position was changed by laying down, sitting, or standing. Initially, the shunt’s pressure was adjusted, and pain medication was given, both of which failed to relieve the pain. A pain physician was consulted, and ultrasound (US) scanning of the left neck region was performed. The ultrasonography showed that the VP shunt passed within the superficial cervical fascia and through the left sternocleidomastoid muscle (SCM) (Figure 2). Additionally, friction of the branches of the superficial cervical plexus and of the greater auricular and lesser occipital nerves caused by the VP shunt was found underneath the lateral border of the SCM (Supplemental Material, Video S1). The irritation of these branches by the shunt catheter was suspected, which could have been the cause of the neck, facial, and scalp pain on the left side, based on the sensory nerve distribution (Figure 3). The blocking and hydro-release of the left superficial cervical plexus were then performed with a combination of 5% glucose solution, lidocaine, and betamethasone. After four series of treatments, each separated by 2 weeks, the neck, facial, and scalp pain vanished, and the frequency of the headaches was substantially reduced. The patient was regularly followed-up in the outpatient department of neurosurgery.

## 3. Discussion

This case is the first to report an inappropriate position of the VP shunt that caused frequent intermittent headaches with left neck pain extending to the left facial and scalp regions. This case also had an unusually delayed presentation. The US scanning performed found that the VP shunt catheter produced the friction of the branches of the superficial cervical plexus and of the greater auricular and lesser occipital nerves. The head and neck pain were successfully relieved with a superficial cervical plexus block and hydro-release.

Complications after VP shunt placement remain a displeasing issue and can occur anywhere along the course of the VP shunt, from the cerebral ventricles to the peritoneal cavity. Despite the development of novel materials for this device, of the design of the device, and of new surgical techniques, complications happen [6]. According to the literature, the overall complication rate of VP shunts ranges from 17% to 52% [7]. The following common complications after VP shunt surgery have been categorized: infections, over-drainage, kinking, disconnection, malpositioning of the catheter tip, catheter obstruction, subdural hematoma, abdominal pseudocyst, and bowel perforation [2,3,4,8]. Catheter obstruction remains the most common cause of shunt malfunction. The most common site of obstruction is the proximal catheter. Various hypotheses have described the reason behind this obstruction. In previous studies, catheters clogged by brain parenchyma, pieces of choroid plexus, or debris, such as blood and proteinaceous fluid, have been mentioned [4]. The second most common cause of malfunction is infection. The risk factors for infection include the following: young age, postoperative CSF leak, glove holes during shunt handling, African American race, public insurance, previous shunt infections, and etiology of intraventricular hemorrhage. Early shunt infection accounts for the majority of the catheter infections which occur within a few weeks or up to several months after VP shunt placement and are often caused by contamination due to normal skin flora during shunt insertion [9]. Late infections happen as well and have been traced to situations of peritonitis, abdominal pseudocyst, bowel perforation, and hematogenous inoculation [4,9]. Patients may present symptoms with headache, lethargy, nausea, or vomiting due to catheter obstruction, as well as fever in the infected ones [4,9]. Besides the common complications mentioned, rare cases associated with VP shunt migration, malpositioning, or dislodging have been reported [6,9,10,11,12]. These rare complications can be encountered either in the immediate post-operative period or during a follow-up appointment [9]. Pseudocyst formation due to VP shunt dislodging in the neck or malpositioning within the abdominal cavity may present with a palpable, distended, and tender mass [9,13,14]. A study reported three pediatric patients with VP shunts who presented chronic right shoulder pain [10]. In these cases, imaging revealed that the distal peritoneal catheter was positioned between the right hemidiaphragm and the liver. And, as the irritation of the diaphragm returns a stimulus via the phrenic nerve to the C3–5 spinal levels, the perceived shoulder pain could have been due to either the stimulation of the supraclavicular nerves of the cervical plexus (C3–4) that innervate the skin over the shoulder or the stimulation of the C5 articular nerves up to the shoulder joint, e.g., from the axillary or suprascapular nerves [10]. C. Lim et al. presented another case with shoulder tip pain following VP shunt insertion due to the malpositioning of the shunt tip, abutting on the left hemidiaphragm [6].

Besides VP shunt catheter-related causes, a broad range of differential diagnoses should be considered in patients with headache and neck pain after VP shunt surgery. These patients should be evaluated thoroughly for any potential complication or underlying conditions, such as meningitis, subdural hematoma, cerebral venous sinus thrombosis, intracranial hypertension due to pseudotumor cerebri, musculoskeletal issues, cervical spine issues, migraine, tension headache, postural headache, or cluster headache. For a differential diagnosis, it is essential to review a patient’s medical record, record their comprehensive history (especially the characteristic of the symptoms), perform a detailed physical examination, and schedule the imaging examinations accordingly. In the present case, the patient experienced headache and neck pain without other neurological symptoms or infection signs and with onset 2 years after VP shunt surgery. Neither shunt infection, shunt obstruction, nor new brain lesions were found, and the symptom did not improve after adjusting the pressure of the VP shunt and administering pain medication. The exact reason as to why the patient’s head and neck pain occurred after a delay of two years is unclear, but the causes of pain were speculated to be related to the VP shunt surgery based on our image findings. This pain was unlikely to be due to the surgery because any surgery-related pain would have happened early after the surgery.

In patients who have undergone VP shunt surgery, head and neck pain occurring after such a long delay has never been reported. This problem could be easily overlooked and symptomatically treated with pain medication only. The detailed image analyses revealed the friction of the branches of the superficial cervical plexus and of the greater auricular and lesser occipital nerves by the VP shunt. The superficial cervical plexus originates from C2 to C4′s anterior primary rami [15]. This plexus branches into the lesser occipital, greater auricular, transverse cervical, and supraclavicular nerves, which emerge from the posterior border of the sternocleidomastoid muscle [16]. When the nerve plexus is irritated, pain could be generated as a cervicogenic headache, neck pain, mandibular pain, para-auricular/ear pain, or even supraorbital pain [15,17]. The distribution of these sensory nerves suggested that the friction of these nerves could have been the cause of the patient’s head and neck pain. This speculation was confirmed by the successful treatment of the patient’s pain with a superficial cervical plexus block and hydro-release.

Malpositioning of the VP shunt is relatively rare; however, once it occurs, patients may suffer from unpleasant sensations and require further management. C. Lim et al. and R. Shane Tubbs et al. reported four cases of patients who experienced shoulder pain after VP shunt placement surgery. Further images proved the malpositioning of the VP shunts. All of them received revision surgery for repositioning the catheter and experienced relief of their shoulder pain postoperatively [6,10]. Saeed Oraee-Yazdani et al. presented another case of malpositioning of a VP shunt. A 17-year-old boy with a history of medulloblastoma surgery and shunt insertion had severe abdominal pain, especially in the right upper quadrant, without rebound tenderness. An abdominal exploration was performed, and they found the tip of the distal catheter located toward the liver, around the falciform ligament. The repositioning of the shunt tip was performed. After the revision surgery, the abdominal pain of the patient was relieved dramatically [18]. In our case, headaches with neck pain were complained by the patient two years after their VP shunt placement surgery. After a series of evaluations, ultrasonography revealed that the VP shunt tube passed within the superficial cervical fascia and through the left SCM muscle, which caused the friction of the branches of the superficial cervical plexus and of the greater auricular and lesser occipital nerves. To solve this situation, we performed the blocking and hydro-release of the left superficial cervical plexus in the patient. After four series of treatments, the pain vanished, and the frequency of the headaches was substantially reduced. The patient had an improved quality of life afterwards and did not receive another surgery for their VP shunt. Among all the complications of a VP shunt, malpositioning is relatively rare and there is no standard management in the current practice. Thoroughly and prudently reviewing a patient’s history and images might be a more effective way of figuring out their fundamental problem. Accordingly, the practitioner could manage the malpositioning of the VP catheter on an individual basis. 

The VP shunt has been widely used in the past few decades and is the most common procedure in patients who need CSF diversion [3,6]. During the placement of a VP shunt, the surgeon needs to make a tunnel from the posterior aspect of the ear, along the SCM, and through the anterior chest wall to the belly. For this purpose, a tunnel at the subcutaneous level is created using a shunt passer, and this procedure largely depends on the surgeon’s experience. Without a tool to confirm the catheter’s location, placing the catheter at levels other than the intended subcutaneous layer is uncommon, but possible. Thus, it is possible that the cause of the shunt’s malpositioning in our subject was due to a surgery error. Alternatively, Rizk et al. [19] suggested that malpositioning of VP shunts occurred more often in younger children because of the thinner subcutaneous tissue in their necks. Previous research mentioned that the ultrasound-guided placement of ventricular catheters in pediatric VP shunt surgery demonstrated an improvement of catheter positioning [20,21]. Thomas Beez et al. compared children receiving ultrasound-guided procedures to matched, historical, freehand controls. They inserted the ventricular catheters under ultrasound-guidance, which is a continuous, real-time visualization of the catheter advancing through the parenchyma and into the desired ventricular compartment. In the study group (n = seventeen), a grade I (optimal) catheter position was achieved in six patients (35%) and a grade II position (contralateral ventricle or contact with ventricular structures) in eleven patients (65%), compared to two (18%) and three patients (27%) in the control group (n = fourteen). This analysis demonstrated an improvement in the accuracy of catheter positioning with ultrasound guidance [21]. Unfortunately, blind tunnelling is regularly performed during VP shunt surgeries in adults [22]. Based on the findings of this and previous cases [19], we advocate that US should be regularly used as a handy tool during surgery to ensure the correct subcutaneous positioning of a shunt’s passer and catheter.

Back to our patient, if the ultrasound-guided treatment had been ineffective and other possible complications had been ruled out, then we would have considered surgery for repositioning their VP shunt.

## 4. Conclusions

In conclusion, we reported a rare case of malpositioning of a VP shunt, which irritated the superficial cervical plexus, resulting in head and neck pain with an unusually long delayed onset after surgery. Nerve block and hydro-release effectively relieved the pain. Based on the findings of this case, the routine use of US for ensuring the correct positioning of a VP shunt is recommended.

## Data Availability

Data sharing not applicable.

## Supplementary Materials

The following supporting information can be downloaded at: https://www.mdpi.com/article/10.3390/medicina59111909/s1, Video S1: Ultrasonography of the friction of branches of the superficial cervical plexus, greater auricular and lesser occipital nerve by the ventriculoperitoneal (VP) shunt was found underneath the lateral border of the sternocleidomastoid muscle (SCM). (White arrow: branches of the superficial cervical plexus; yellow circle: VP shunt tube).
